# Methane biohydroxylation into methanol by *Methylosinus trichosporium* OB3b: possible limitations and formate use during reaction

**DOI:** 10.3389/fbioe.2024.1422580

**Published:** 2024-08-26

**Authors:** Héloïse Baldo, Azariel Ruiz-Valencia, Louis Cornette de Saint Cyr, Guillaume Ramadier, Eddy Petit, Marie-Pierre Belleville, José Sanchez-Marcano, Laurence Soussan

**Affiliations:** Institut Européen des Membranes, IEM–UMR 5635, Univ. Montpellier, ENSCM, CNRS, Montpellier, France

**Keywords:** biohydroxylation, *Methylosinus trichosporium* OB3b, methanol production, formate, limitations

## Abstract

Methane (CH_4_) hydroxylation into methanol (MeOH) by methanotrophic bacteria is an attractive and sustainable approach to producing MeOH. The model strain *Methylosinus trichosporium* OB3b has been reported to be an efficient hydroxylating biocatalyst. Previous works have shown that regardless of the bioreactor design or operation mode, MeOH concentration reaches a threshold after a few hours, but there are no investigations into the reasons behind this phenomenon. The present work entails monitoring both MeOH and formate concentrations during CH_4_ hydroxylation, where neither a gaseous substrate nor nutrient shortage was evidenced. Under the assayed reaction conditions, bacterial stress was shown to occur, but methanol was not responsible for this. Formate addition was necessary to start MeOH production. Nuclear magnetic resonance analyses with ^13^C-formate proved that the formate was instrumental in regenerating NADH; formate was exhausted during the reaction, but increased quantities of formate were unable to prevent MeOH production stop. The formate mass balance showed that the formate-to-methanol yield was around 50%, suggesting a cell regulation phenomenon. Hence, this study presents the possible physiological causes that need to be investigated further. Finally, to the best of our knowledge, this study shows that the reaction can be achieved in the native bacterial culture (*i.e.*, culture medium containing added methanol dehydrogenase inhibitors) by avoiding the centrifugation steps while limiting the hands-on time and water consumption.

## 1 Introduction

Atmospheric methane (CH_4_) is the second largest contributor to the greenhouse effect after carbon dioxide (CO_2_); its global warming potential is known to be 84-fold higher than that of CO_2_ over 20 years and 28-fold over a century (Pachauri et al., 2015). CH_4_ is the main component of natural gas and biogas and can be valorized directly as an energy source or used as a raw material to produce valuable chemicals such as methanol (MeOH). This latter molecule has garnered growing interest as MeOH can be used as a precursor for plastic and fuel production. Since methanol is liquid at room temperature, it is safer and more convenient to store and transport than pressured gas. Industrial processes that convert CH_4_ to MeOH currently involve natural gas steam reforming followed by Fisher–Tropsch synthesis on the generated syngas. Several conversion processes exist, but these generally require pressures of around 30 bar and temperatures up to 1,000°C (Aasberg-Petersen et al., 2011; Soussan et al., 2016; Lee, 2019), which make them energy intensive. Moreover, these methods only allow partial selectivity and result in significant CO_2_ emissions (Soussan et al., 2016; Bjorck et al., 2018).

**FIGURE 1:**
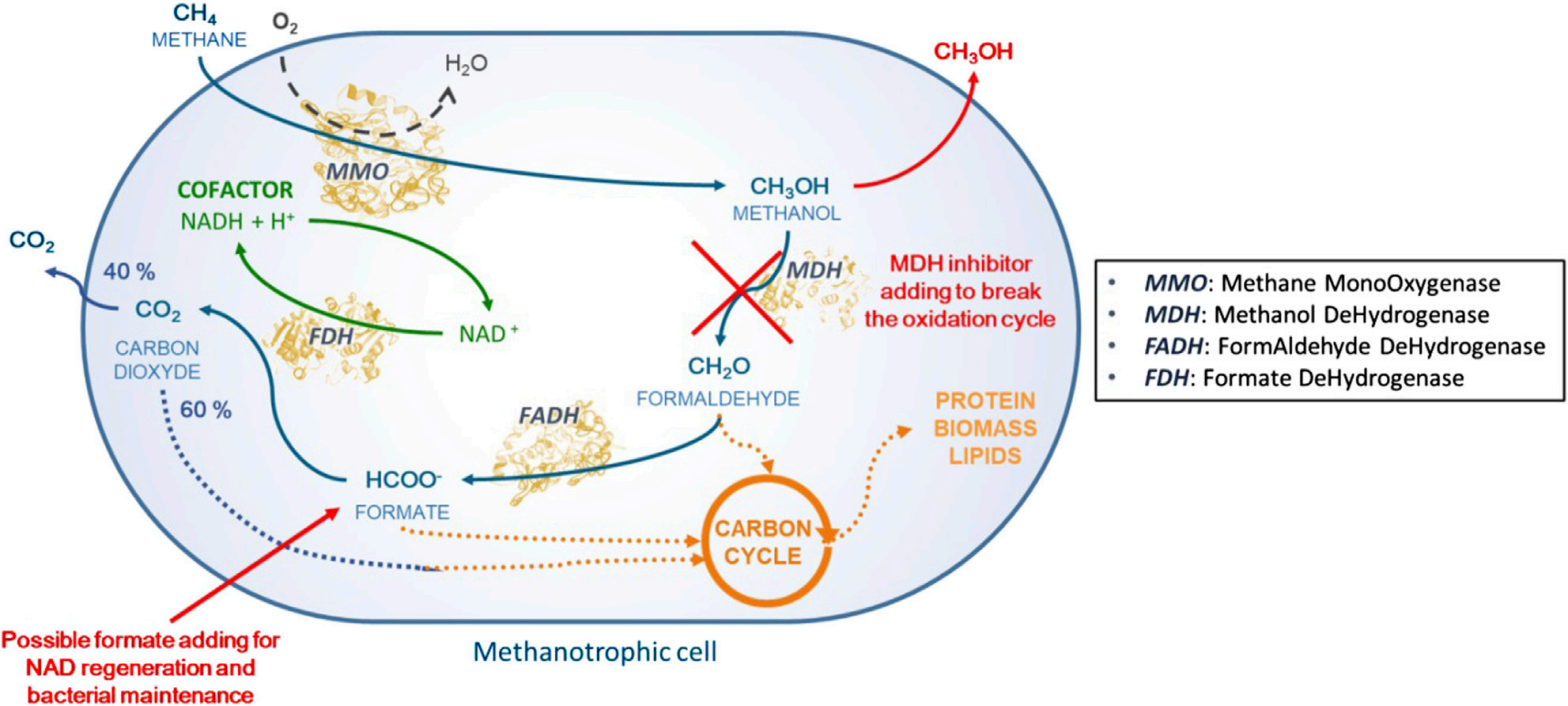
General metabolic pathway for CH_4_ assimilation in methanotrophic bacteria.

Biological conversion of CH_4_ into MeOH (*i.e.*, biohydroxylation) by methanotrophic bacteria appears to be a promising approach for more sustainable CH_4_ valorization. Biohydroxylation is a highly selective one-pot reaction carried out under physiological conditions; it exhibits favorable carbon balance and generates water as the only byproduct. *Methylosinus trichosporium* OB3b is a model mesophilic non-pathogenic strain that grows at around 30°C under atmospheric pressure, whose hydroxylating abilities are well-known (Whittenbury et al., 1970). Methanotrophic central carbon metabolism utilizes CH_4_ as the sole energy and carbon source, where CH_4_ is fully oxidized to CO_2_ through sequential enzymatic oxidations. As shown in Figure 1, the enzyme methane monooxygenase (MMO) is responsible for CH_4_ uptake and oxidation into MeOH as per Equation 1:

$${CH}_{4}+O_{2}+NADH+H^{+}\to \;{CH}_{3}OH+{NAD}^{+}+H_{2}O$$ (1)

Methanol dehydrogenase (MDH) then oxidizes MeOH into formaldehyde, which is later converted into formate by formaldehyde dehydrogenase (FADH). Formate dehydrogenase (FDH) then catalyzes the final oxidation of formate into CO_2_, with concomitant NAD^+^ regeneration into NADH. Thereafter, more than 60% of the CO_2_ produced is assimilated into the bacterial biomass (Yang et al., 2013). Thus, FDH allows regeneration of the reducing equivalents required for the catalytic activity of MMO (Figure 1).

*M. trichosporium* OB3b synthesizes two MMO forms, namely soluble MMO (sMMO) and particulate MMO (pMMO), depending on the Cu^2+^ concentration (Sirajuddin and Rosenzweig, 2015). Both enzymes catalyze the same oxidation reaction, but pMMO is reported to have greater affinity toward CH_4_ (Sirajuddin and Rosenzweig, 2015; Bjorck et al., 2018). pMMO is not stable outside the cell membrane, which explains why whole-cell bacteria are still preferred over the isolated enzyme to catalyze CH_4_ oxidation into MeOH (Sirajuddin and Rosenzweig, 2015; Soussan et al., 2016). MeOH production has been shown to be exogenous, *i.e.*, MeOH is accumulated in the external medium (Pen et al., 2014). Thus, MeOH accumulation in whole-cell methanotrophs relies on inhibiting MDH (Figure 1). Several MDH inhibitors have been reported to date, such as high phosphate concentrations, NaCl, ethylenediaminetetraacetic acid (EDTA), MgCl_2_, and cyclopropanol. Previous studies have evidenced the synergistic effects of NaCl and EDTA (Pen et al., 2014) in ensuring efficient MeOH production.

As depicted in Figure 1, MDH inhibition interrupts the successive oxidations of MeOH, thus causing collateral damage to the downstream FDH enzyme. This deprives FDH of its substrate, stopping both formate oxidation and NAD^+^ reduction. The addition of sodium formate (NaCOOH) as an external electron donor is therefore necessary to ensure NADH regeneration by FDH, as expressed by Equation 2.

$${NAD}^{+}+{HCOO}^{-}\to NADH+{CO}_{2}$$ (2)

Formate also plays a key role in the metabolism of *M. trichosporium* OB3b (Figure 1); for example, it enters the tetrahydrofolate pathway to feed the downstream serine cycle (Matsen et al., 2013) and contributes to general bacterial metabolism. Previous studies have shown that an initial sodium formate concentration of 20 mmol/L enhances MeOH production (Lee et al., 2004; Duan et al., 2011). MeOH production balance is thus given by Equation 3:

$${CH}_{4}+O_{2}+{HCOO}^{-}+H^{+}\to {CH}_{3}OH+{CO}_{2}+H_{2}O$$ (3)

To the best of our knowledge, the highest reported methanol productivity by *M. trichosporium* OB3b was 270 mg MeOH/g_dry cell_/h under batch conditions (Pen et al., 2014). The production kinetics of MeOH are usually divided into three phases, where MeOH first increases linearly, then slows down, and finally stabilizes around a maximum value corresponding to a concentration plateau. This MeOH production plateau is reported to occur at around 10 h for all methanotrophic strains, bioreactor designs, and operation modes tested (Mehta et al., 1991; Lee et al., 2004; Kim et al., 2010; Duan et al., 2011; Pen et al., 2014; Farhan Ul Haque et al., 2016; Mardina et al., 2016; Taylor et al., 2018; Ito et al., 2021; Kulkarni et al., 2021; Bo et al., 2023).

The present work seeks to understand the reasons behind the stop of MeOH accumulation so as to extend the MeOH production time. The potential limitations that could cause MeOH production stop were consequently studied herein. First, the MeOH generation pathway was evaluated through ^13^C-labeled assays. Then, the gaseous substrates, nutrients, or external electron donor limitations were considered. Methanol toxicity on the bacteria was also assessed. Finally, formate use was further elucidated by investigating the relationships between the formate and MeOH concentrations.

## 2 Material and methods

Unless noted otherwise, all chemicals listed hereafter were supplied by Sigma-Aldrich, France. Their purity grade was at least that specified by the American Chemical Society. Ultrapure water was produced with a Milli-Q system (Millipore), and ambient air was used wherever required. CH_4_ (purity: 99.5%) was provided by Linde, France.

### 2.1 Bacterial strain and cultivation conditions

*M. trichosporium* OB3b (NCIMB 11131) was rehydrated and cultured in a modified medium containing nitrate mineral salts (NMS), as described by Pen et al. (2014). The NMS base medium contained 1.06 g/L of KH_2_PO_4_, 4.34 g/L of Na_2_HPO_4_∙12H_2_O, 1.70 g/L of NaNO_3_, 0.34 g/L of K_2_SO_4_, and 0.074 g/L of MgSO_4_∙7H_2_O, which was then steam-sterilized; the pH was adjusted to 7.0 ± 0.1 with 0.1 M HCl or NaOH. In parallel, solutions of 1000× copper (0.798 g/L of CuSO_4_), iron (11.2 g/L of FeSO_4_∙7H_2_O), and trace elements (7.0 g/L of CaCl_2_∙2H_2_O, 0.57 g/L of ZnSO_4_∙7H_2_O, 0.445 g/L of MnSO_4_∙H_2_O, 0.124 g/L of H_3_BO_3_, 0.096 g/L of Na_2_MoO_4_∙2H_2_O, 0.096 g/L of CoCl_2_∙6H_2_O, and 0.166 g/L of KI) were prepared separately and sterilized using 0.22 µm polyethersulfone (PES) sterile syringe filters (Branchia). Each solution was then supplied at 1 mL/L to the base NMS medium.

The strain was cultured in sealed glass vials under a CH_4_/air atmosphere (1:4 *v/v*) and incubated at 30°C under rotation at 160 rpm (Unimax 1010, Heidolph). Before each liquid subculture and methanol production assay, the bacterial suspensions were plated on agar plates prepared with 25 g/L of Miller’s lysogeny broth (LB) and 15 g/L of microbiological agar before being incubated at 30°C to check for the absence of heterotrophic contamination. The bacterial cultures were maintained on the NMS agar plates grown under the CH_4_/air (1:4 *v/v*) atmosphere at 30°C. The plates were subcultured once a month.

### 2.2 Methanol production assays in batch mode

The methanol production assays were carried out in either the reference or modified reaction medium (RM). The reference RM was composed of 1.07 g/L of KH_2_PO_4_, 1.80 g/L of Na_2_HPO_4_∙12 H_2_O, 5.844 g/L of NaCl, 1.36 g/L of CHNaO_2_, and 0.292 g/L of EDTA, whose pH was adjusted to 7.0 ± 0.1 with 0.1 M HCl or NaOH (Kim et al., 2010; Pen et al., 2014). In some cases, the formate concentration was changed upon comparison with the reference RM. Some experiments were also carried out in the RM by replacing ^12^C-formate with ^13^C-formate and in the NMS medium supplemented with the same NaCl, CHNaO_2_, and EDTA concentrations as in the reference RM. The NMS medium could be either freshly prepared or native, *i.e.,* a culture medium in which the bacterial cells had grown and already exhausted a portion of the supplied nutrients.

Bacterial cells cultured in the NMS medium were collected during exponential growth and centrifuged at 4°C and 3,893*g* for 20 min. The NMS medium was then discarded and cell pellets were resuspended in an equivalent volume of fresh reference or test RM. In all assays, the dry cell concentration of the resting cell suspension reached 0.25 ± 0.1 g_dry cell_/L (Section 2.3), 6 mL of the resting cell suspension were then transferred in 60 mL gas-tight sealed vials. For each experiment, a set of identical vials was filled with the same bacterial suspension and incubated. MeOH accumulation was then induced by filling the headspace with a mixture of CH_4_/air (1:4 *v/v*) that was previously filtered using autoclaved 0.20 µm polytetrafluoroethylene (PTFE) filters (Sartorius). The reactors were incubated at 30°C under 160 rpm rotation (Unimax 1010, Heidolph), and the reactors were sampled after different reaction times.

The gaseous substrate limitations were investigated in some of the experiments. Here, MeOH production was carried out in the reference RM, and the gaseous headspace was renewed twice during the reaction by pumping it out and refilling with a mixture of CH_4_/air (1:4 *v/v*) at t = 6 h and t = 24 h. For each MeOH production experiment, two independent controls (*i.e.*, operated under the reference conditions) were implemented against two independent test reactors. MeOH was also supplied to the RM initially to assess methanol toxicity in one assay.

### 2.3 Analyses

Samples containing 2 mL of each bacterial suspension were used for direct pH and optical density (**O**D) measurements (Section 2.3.1). The remaining suspension volumes were immediately transferred to 2 mL safe-lock Eppendorf tubes. Samples dedicated to further formate analyses (Section 2.3.2) were stored as pelleted suspensions, while those used for ulterior MeOH quantifications were centrifuged at 4°C and 3,893*g* for 4 min to eliminate the cell pellets before storage. All samples were stored at −20°C until analyses.

#### 2.3.1 pH and OD monitoring

The biomass concentrations were estimated through OD measurements at 600 nm (OD_600nm_) with a UV-VIS 2401PC spectrophotometer (Shimadzu) using the following correlation (Equation 4) (Pen et al., 2014):

$$\left[dry\;biomass\right]\left(g_{dry\;cell}/L\right)=0.322*\;{OD}_{600nm}$$ (4)

The pH was monitored during the reaction with a C831 pH meter (Consort).

#### 2.3.2 Methanol and formate quantifications by gas chromatography mass spectrometry (GC-MS)

Methanol and formate were quantified using a gas chromatography (Trace 1300, Thermo Scientific) mass spectrometry (ISQ 7000, Thermo Scientific) system. The electronic impact source was operated at 70 eV, and both analyses required a Rt-Q-Bond Plot capillary column (30 m × 0.25 mm ID, Restek) and helium at 1.2 mL/min as the carrier gas. 0.5 µL of the sample volume was introduced into the injector at 250°C, and a split ratio of 100 was applied to quantify formate and methanol.

Methanol accumulation kinetics was monitored in the reaction supernatants. High-performance liquid chromatography (HPLC)-grade pure ethanol (Carlo Erba) was added to the thawed reaction supernatant as the internal standard at a final concentration of 29.6 mg/L. This method involved prior heating at 90°C, followed by two successive temperature gradients of 10°C/min up to 150°C (hold time of 3 min) and 20°C/min up to 200°C (hold time of 10 min).

The formate consumption kinetics was monitored in the whole bacterial suspensions using a previously described method (Ruiz-Valencia et al., 2020), where the samples underwent a derivatization treatment after thawing. Methanol esterification on the formate was carried out with the addition of pure MeOH and H_2_SO_4_ (Equation 5), and the methyl formate produced was quantified by GC-MS. HPLC-grade pure acetonitrile was added as the internal standard at a final concentration of 17.47 mg/L. This method involved prior heating at 40°C, followed by two successive temperature gradients of 10°C/min up to 150°C (hold time of 3 min) and 20°C/min up to 200°C (hold time of 10 min).

$${CH}_{3}OH+HCOO^{-}+H^{+}\to HCOO{CH}_{3}+H_{2}O$$ (5)

The ^12^C and ^13^C-labeled MeOH and formate were discriminated according to the following mass-to-charge ratios (m/z): 31 for ^12^C-MeOH, 33 for ^13^C-MeOH, 60 for ^12^C-methyl formate, and 61 for ^13^C-methyl formate.

#### 2.3.3 ^13^C-labeled formate monitoring with nuclear magnetic resonance (NMR)

^13^C spectra were recorded at 298 K on a Bruker Avance III 500 NMR spectrometer equipped with a 5 mm ^1^H/X BBO Helium cryoprobe. All data were processed with the Bruker Topspin 3.6.2 software. The ^13^C UDEFT sequence was used (NS = 1024 scans), and all NMR analyses were conducted in duplicate in 5-mm-diameter tubes. A control was also implemented with a non-inoculated reaction medium to determine the chemical shift of ^13^C-labeled sodium formate (δ = 171.02 ppm). 900 µL of each resting cell suspension was sampled at t = 0 h as the control, followed by sampling at t = 4 h, t = 20 h, and t = 48 h. 100 μL of D_2_O was added to each NMR tube before analysis. The concentrations of ^12^C-MeOH and formate must reach at least 500 mg/L to be detected by ^13^C NMR. By enriching the compounds with ^13^C (instead of ^12^C), the NMR carbon acquisition sensitivity can be increased to more than 90-fold (Ruiz-Valencia et al., 2020, 2024). The lower detection limit of NMR for ^13^C-MeOH and ^13^C-formate was indeed about 6 mg/L, as shown in previous studies (Ruiz-Valencia et al., 2020, 2024).

## 3 Results and discussion

### 3.1 MeOH production and reproducibility

**FIGURE 2:**
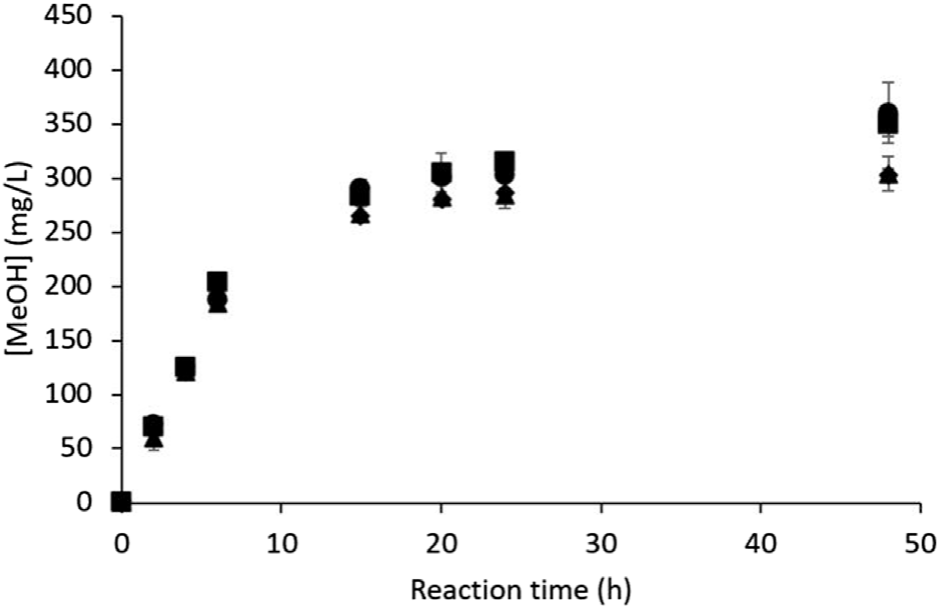
MeOH production kinetics by *M. trichosporium* OB3b in the batch mode under the reference conditions (results of four independent assays).

The ability of *M. trichosporium* OB3b to oxidize CH_4_ into MeOH was assessed under the reference conditions (see Section 2.2), which were chosen according to a previous study (Pen et al., 2014) that showed that this RM composition produced the best MeOH hydroxylation performances over those relying on high phosphate concentrations for MDH inhibition (Mehta et al., 1991; Duan et al., 2011). The MeOH concentration ([MeOH], mg/L) was monitored using four independent assays (Figure 2). Each independent assay was conducted with a different subculture (one month maximum between two consecutive subcultures) and was duplicated with the same bacterial suspension. The pH and dry biomass concentration ([dry biomass], g_dry cell_/L) were measured at the beginning and end of the experiment.

The MeOH production kinetics were almost identical in all cases, where MeOH was produced quasilinearly during the first 6 h until reaching a concentration of 190 ± 10 mg/L (Figure 2). MeOH accumulation continued thereafter but slowed significantly until 20 h, at which point the concentration was 292 ± 14 mg/L; thereafter, the production evolved by very little up to 48 h (Figure 2), with the MeOH concentration either stabilizing (hence achieving a plateau around 303 ± 10 mg/L) or accumulating very slightly (reaching a maximum of 355 ± 19 mg/L). Nevertheless, the final MeOH concentration had the same order of magnitude for all four experiments. These results are in accordance with previous studies (Pen et al., 2014; Pen et al., 2016) carried out under similar conditions.

The differences observed in the final concentrations of MeOH may be attributed to various factors, mostly biological variability and evolution of the specific activity of *M. trichosporium* OB3b along the subcultures. Consequently, the control kinetics under the reference conditions were always implemented against the test conditions in the remaining studies. The pH increased from 6.98 ± 0.00 at t = 0 h to 7.74 ± 0.04 at t = 48 h; this moderate-to-medium alkalinization is consistent with Equation 1 since MeOH production requires consuming the molar equivalent of protons to oxidize CH_4_ (1:1 stoichiometry, Equation 3). Whittenbury et al. (1970) noted that all of their isolated methanotrophic strains, including *M. trichosporium* OB3b, grew well on a broad pH range of 5.8–7.4. The pMMO extraction protocols often involve buffers with pH of around 7.5 (Lieberman et al., 2003). The maximum pH value of 7.75 ± 0.01 obtained herein was thus not considered to be harmful to the bacterial physiology. To evaluate this assumption, *M. trichosporium* OB3b growth tests were conducted at pH = 7.0 and pH = 7.85; no significant differences were observed (Supplementary materials), confirming that such moderate-to-medium alkalinization had no deleterious effects.

The OD_600nm_ was monitored at the same reaction time at which the MeOH concentration was quantified. The OD_600nm_ measurement is known to be mainly sensitive to whole cells, *i.e.*, delimited by biological membranes (Beal et al., 2020). Thus, a significant decrease in the OD_600nm_ value means that cell lysis (and death) occurred. On the contrary, increasing values of OD_600nm_ evidence cell proliferation, whereas constant OD_600nm_ values imply constant cell concentrations. The relevance of using OD_600nm_ to monitor the *M. trichosporium* OB3b cell concentration was thus assessed. A correlation between the OD_600nm_ value and dry cell concentration was established previously (Equation 4; Section 2.3.1); an additional correlation between the OD_600nm_ values and concentrations of fresh cultivable cells ([*cultivable cells*], CFU/mL) was obtained for *M. trichosporium* OB3b based on four observations, with a linear determination coefficient (R^2^) of 0.9506.

$$\left[cultivable\;cells\right]\left(CFU/mL\right)=1.21\;10^{6}*\;{OD}_{600\;nm}$$ (6)

The OD_600nm_ value is thus an appropriate tool for monitoring *M. trichosporium* OB3b cell concentration. Nevertheless, the cell viability and cultivability are likely to evolve over time, which renders the correlation between OD_600nm_ and dry biomass concentration more reliable. In this study, OD_600nm_ was converted into the dry biomass concentration in accordance with Equation 4. The dry biomass within the reactor generally remained constant, beginning at 0.331 ± 0.004 g_dry cell_/L at t = 0 h immediately after cell pellet resuspension in the RM and ending at 0.318 ± 0.003 g_dry cell_/L at t = 48 h (which is less than 5% variation from the initial dry biomass concentration). Two hypotheses may be used to explain such results: bacterial death and proliferation occurred simultaneously with identical rates or the dry cell concentration remained steady. Knowing that the bacteria could be grown in the reaction medium devoid of MDH inhibitors in the same way as that in the NMS culture medium (reaching similar OD_600nm_ values of around 0.292 ± 0.002 g_dry cell_/L within 22 h under both conditions), this means that the presence of MDH inhibitors was efficient enough to prevent methane uptake and the subsequent biomass synthesis pathway. Therefore, the dry biomass was constant under the reaction conditions tested, and the equilibrium between bacterial growth and death was ruled out. It is worth noting that very little bacterial lysis occurred despite the stressful conditions experienced by the bacterial cells (*i.e.*, disruption of CH_4_ assimilation pathway, Figure 1).

### 3.2 Elucidating the MeOH production pathway

According to Figure 1, MeOH is an intermediate metabolite in the methanotrophic core carbon metabolism. FDH, FADH, and MDH enzymes are known to be reversible, *i.e.*, they can either oxidize the CH_4_-deriving molecules or reduce the CO_2_-deriving molecules (Xin et al., 2007; Sahoo et al., 2022; Sahoo et al., 2023), whereas MMO is an irreversible enzyme that is only able to oxidize CH_4_ into MeOH (Pen et al., 2016). MeOH could therefore result from CH_4_ oxidation catalyzed by MMO or from CO_2_/formate/formaldehyde serial reductions by FDH, FADH, and MDH, respectively. Therefore, the initial addition of sodium formate as an external electron donor could also be a carbon source for MeOH production.

**FIGURE 3:**
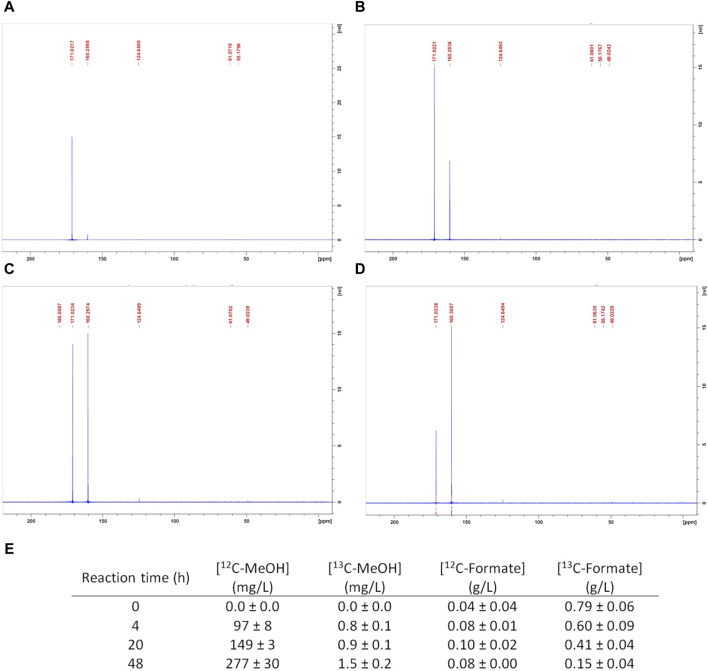
CH_4_ hydroxylation by *M. trichosporium* OB3b in the batch mode under the reference conditions with ^13^C-formate: nuclear magnetic resonance spectra at **(A)** t = 0 h, **(B)** 4 h, **(C)** 20 h, and **(D)** 48 h; **(E)** gas chromatography mass spectrometry quantifications of ^12^C-MeOH, ^13^C-labeled MeOH, ^12^C-formate, and ^13^C-labeled formate.

To discriminate between both of the possible MeOH origins, an experiment was carried out under the reference conditions with ^13^C-labeled formate. Whole-cell bacterial suspensions were sampled and analyzed at t = 4 h (during linear MeOH production), t = 20 h (beginning of MeOH production slowdown), and t = 48 h (MeOH accumulation stop). The ^13^C-NMR analyses were complemented by quantifications of the ^12^C- and ^13^C-labeled MeOH and formate through GC-MS. The corresponding ^13^C-NMR spectra are presented in Figure 3 along with the GC-MS results. The sample spectra exhibit two significant peak variations over t = 0 h to t = 48 h, whose corresponding chemical shifts (δ) were detected as 171.02 and 160.2 ppm. An additional signal with a chemical shift of 124.6 ppm was visible at t = 4 h, which remained at a low intensity thereafter.

A control tube containing the non-inoculated ^13^C-formate RM was analyzed previously. Its NMR spectrum showed a single peak with a chemical shift of 171.02 ppm without any background noise (data not shown). This 171.02 ppm signal was attributed to ^13^C-formate, and the intensity corresponding to this peak decreased from t = 0 h to t = 48 h (Figures 3A–D), suggesting ^13^C-formate uptake. This consumption was confirmed by GC-MS. The ^13^C-formate concentration indeed diminished from 0.79 ± 0.06 g/L at t = 0 h to 0.15 ± 0.04 g/L at t = 48 h (Figure 3E). The ^12^C-formate concentration generally remained constant at a low value (around 0.08 g/L, Figure 3E), meaning that there was no ^12^C-formate production. Therefore, ^12^C-formate was initially present in the reaction mixture, and the mean ^12^C-formate concentration represented no more than 10% of the initial ^13^C-formate concentration.

Meanwhile, a peak with a chemical shift of 160.2 ppm was observed at 4 h (Figure 3B), whose signal intensity increased up to 48 h (Figures 3B–D). Previous studies have already identified the corresponding molecule as the ^13^C-labeled hydrogenocarbonate ion (H^13^CO_3_^−^) (Ruiz-Valencia et al., 2020, 2024). According to the pKa value of H_2_O,CO_2_/HCO_3_^−^, H^13^CO_3_^−^ is the main solubilized form of carbon dioxide in the pH range of 7–8 (*i.e.*, pH variations observed during reaction under the reference condition). Furthermore, a peak corresponding to a chemical shift of 124.6 ppm was significantly observed at t = 4 h (Figure 3B). Previous studies have already attributed this peak to ^13^C-labeled CO_2_. Its intensity was inferior to the peak attributed to H^13^CO_3_^−^ and generally remained constant up to t = 48 h (Figures 3B–D), confirming that H^13^CO_3_^−^ was the prevalent form of ^13^C-labeled CO_2_ under these operating conditions.

The NMR spectra (Figures 3A–D) did not exhibit any peaks at 50.1 or 50.2 ppm, which are the corresponding chemical shifts of ^13^C-MeOH (Cornish et al., 1984; Mason et al., 1987). However, the GC-MS results show that ^12^C-MeOH was produced at a maximum concentration of 277 ± 30 mg/L at 48 h (Figure 3E), as is usually obtained under the reference reaction conditions. In addition, the ^13^C-MeOH concentration remained negligible and never exceeded 1.5 ± 0.2 mg/L, accounting for 0.5% of the ^12^C-MeOH at t = 48 h (Figure 3E). ^13^C-MeOH did not exceed its natural occurrence and could therefore not be observed in the NMR spectra as well as unlabeled MeOH produced, whose concentration was below 500 mg/L (see Section 2.3.3).

The absence of the ^13^C-associated peak apart from those associated with ^13^C-formate consumption and ^13^CO_2_ production (mainly under its basic form H^13^CO_3_^−^) confirms that the cells likely used ^13^C-formate as the external electron donor for NADH regeneration by FDH, according to Equation 2 and Figure 1. Despite some studies reporting that *M. trichosporium* strains (including OB3b) were able to produce MeOH by reversing their CH_4_ oxidation pathways (Xin et al., 2007; Sahoo et al., 2022; Sahoo et al., 2023), formate was not reduced to ^13^C-MeOH under our operating conditions. The MeOH produced was solely a result of CH_4_ oxidation by MMO. Moreover, these data confirm that MMO is an irreversible enzyme as ^13^CH_4_ would have been generated otherwise.

Nevertheless, as observed in Figure 2, MeOH production decreased with time and tended to level off at a final concentration of around 300 mg/L. A previous study carried out at our laboratory excluded MeOH inhibition on the MMO enzyme (Pen et al., 2016). Indeed, fresh RM renewals during the reaction at 4.5 h and 22 h led to a final MeOH quantity that was not significantly different from that obtained under the reference conditions, *i.e.,* without RM renewal (Pen et al., 2016). Thus, MeOH production stop could be a result of gaseous substrates and/or nutrient limitations as well as electron donor shortage preventing NADH regeneration. These hypotheses were also investigated in this study, as detailed below.

### 3.3 Gaseous substrate limitations

Methane oxidation into MeOH requires CH_4_ and O_2_ as substrates at molar equivalents (1:1 stoichiometry, Equation 1). The reactors were filled with a mixture of CH_4_/air (1:4 *v/v*) at t = 0 h. Since the ambient atmosphere contains around 80% N_2_ and 20% O_2_, the injected gas mixture allowed an approximately equimolar initial CH_4_/O_2_ ratio in the vials. The theoretical calculations based on the ideal gas law show that the initial gas quantities in the vials could result in almost 6-fold superior MeOH concentrations (1.86 g/L of MeOH) compared to the obtained results (*i.e.*, MeOH plateau around 300 mg/L, Figure 2). Pen et al. (2014) previously detailed similar calculations in their study. Thus, CH_4_ and O_2_ are not assumed to be limiting.

**FIGURE 4:**
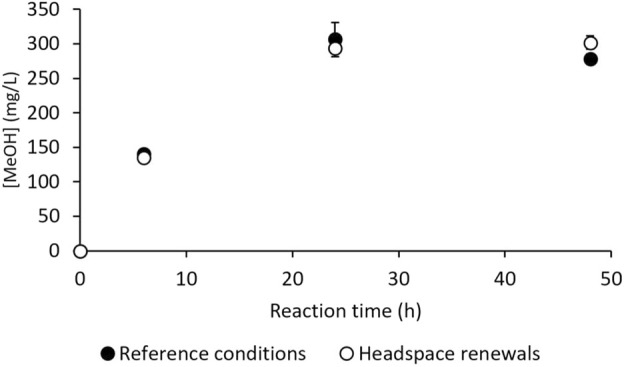
MeOH production by *M. trichosporium* OB3b in the batch mode under the reference conditions (filled circles) and with headspace renewals at 6 and 24 h (empty circles).

*M. trichosporium* OB3b is an obligate aerobic methanotroph, and its metabolism may use O_2_ in competing metabolic pathways, such as the electron transport chain, to generate energy during respiration. Moreover, O_2_ solubility in aqueous media at 30°C is about 10-fold lower than that of CH_4_, making it the most suitable candidate for substrate limitation under batch conditions (Perry and Green, 1998; Pen et al., 2014). A practical assay was thus conducted to check the absence of gaseous substrate limitations. In this test, the headspace gases in the vials were pumped out and replaced with a fresh mixture of CH_4_/air (1:4 *v/v*) at 6 h (end of the linear MeOH production phase, Figure 2) and 24 h (near the end of MeOH production, Figure 2). An assay was also conducted under the reference conditions (*i.e.*, without any gas renewal) simultaneously; these results are given in Figure 4. They show that headspace renewal during the reaction had no effect on the quantity of MeOH produced or on the production kinetics, highlighting that the MeOH production plateau is not due to any gaseous substrate limitation during the reaction.

### 3.4 Nutrient limitations

**FIGURE 5:**
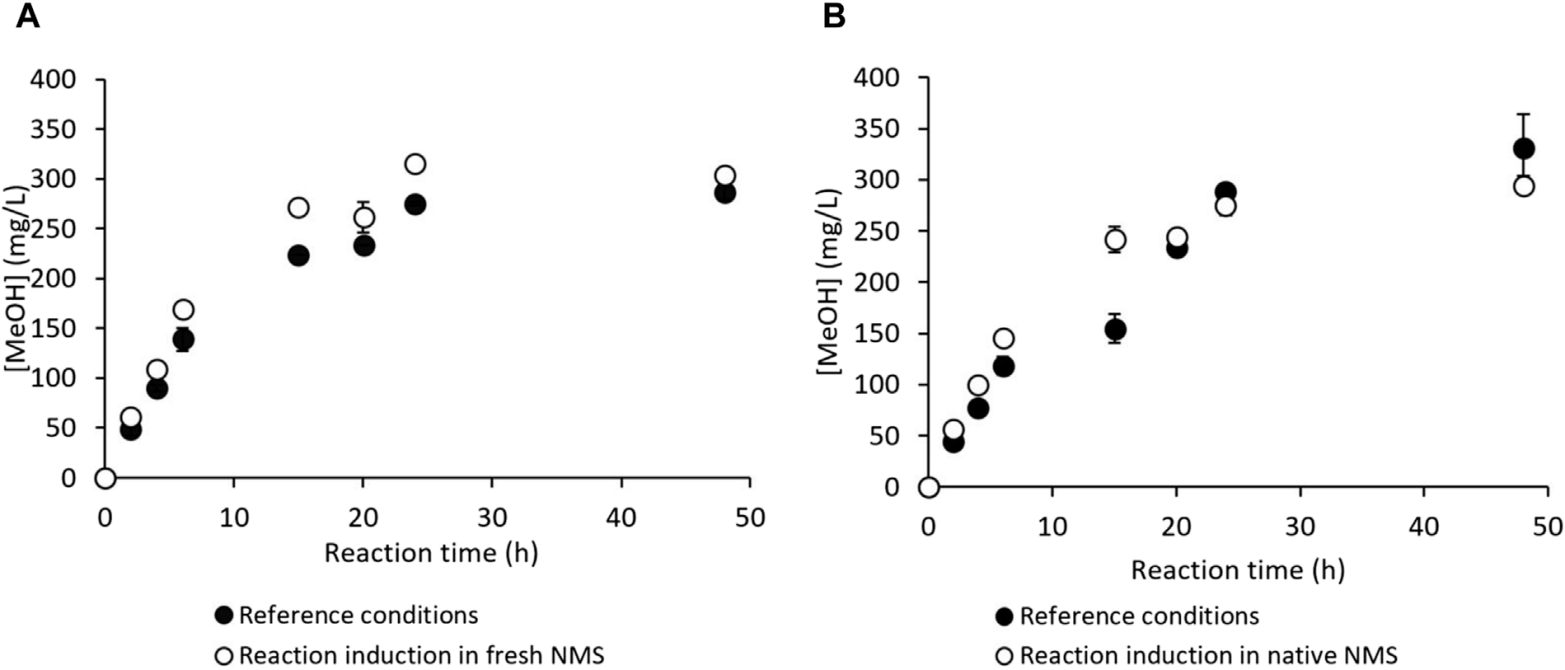
MeOH production by *M. trichosporium* OB3b in the nitrate mineral salt (NMS) culture medium in the batch mode **(A)** under the reference conditions (filled circles) and in fresh NMS supplemented with formate and methanol dehydrogenase (MDH) inhibitors, *i.e.*, NaCl and EDTA (empty circles); **(B)** under the reference conditions (filled circles) and in the native NMS culture medium supplemented with formate and MDH inhibitors (empty circles).

The NMS culture medium is formulated to support methanotroph proliferation, which implies normal carbon metabolism and optimal MMO catalytic activity. In particular, it contains nitrates (NO_3_^−^) that act as nitrogen sources and diverse micronutrients. On the contrary, the RM composition is limited to phosphates as the buffering agents, NaCl and EDTA as the MDH inhibitors, and sodium formate as an external electron donor for NADH regeneration (Section 2.2). A potential nutrient shortage could be detrimental to bacterial physiology and/or affect their ability to maintain sufficient and functional MMO stock during reaction. Therefore, MeOH production in the richest medium suitable for methanotroph metabolism was assayed by adding the reference NaCl, EDTA, and formate quantities to fresh NMS medium (Figure 5A). In this case, the fresh RM and amended fresh NMS ionic strengths had the same orders of magnitude (around 0.1–0.2 mol/L). Another assay was conducted with the native NMS directly, *i.e.*, the culture medium in which the bacterial cells had grown and already exhausted a portion of the nutrients (Figure 5B). This spent culture medium containing bacterial cells (*i.e.*, bacterial suspension in exponential growth) was supplied with a small volume of a solution containing concentrated formate and MDH inhibitors to achieve the same concentration as in the reference RM without inducing significant dilution (Section 2.2). The trials under the reference conditions described in Section 2.2 were always implemented in parallel (Figures 5A, B).

Figure 5A shows no improvement in MeOH production with the fresh NMS supplemented with formate and MDH inhibitors compared to the reference RM. Thus, *M. trichosporium* OB3b does not appear to experience any nutrient shortage during reaction. The reference conditions showed very little dry biomass evolution from 0.233 ± 0.001 g_dry cell_/L at t = 0 h to 0.243 ± 0.006 g_dry cell_/L at t = 48 h. The dry biomass evolved similarly to that in NMS supplemented with formate and MDH inhibitors (*i.e.*, from 0.229 ± 0.001 g_dry cell_/L at t = 0 h to 0.265 ± 0.002 g_dry cell_/L at t = 48 h), indicating no bacterial growth during the reaction and appropriate MDH inhibition.

No significant difference in MeOH production was evidenced between the reference and native NMS conditions (Figure 5B). Although this procedure does not extend the MeOH production time, it has several practical advantages. The culture phase suspensions no longer require centrifugation to discard the spent NMS or resuspension of the bacterial pellet in fresh RM. Therefore, it is more convenient, has lower contamination risk, reduces the hands-on working time, and limits water consumption due to the preparation of large RM volumes. After verifying the absence of substrate and nutrient limitations, this study focused on the potential toxicity of methanol against the bacteria.

### 3.5 Methanol toxicity

There are extensive investigations in literature regarding MeOH toxicity on *M. trichosporium* OB3b growth under culture conditions. The reported toxicity threshold concentrations for *M. trichosporium* OB3b vary strongly depending on the source (Whittenbury et al., 1970; Best and Higgins, 1981; Adegbola, 2008). Some studies have reported severe toxicity with concentrations as low as 0.01% (w/v) equivalent to 0.1 g/L (Whittenbury et al., 1970), while others have evidenced possible growth up to 4% (v/v) (equivalent to 31.6 g/L) after adaptation to MeOH (Best and Higgins, 1981). Other researchers have shown growth rate decreases for MeOH ranging from 0.3 g/L to 0.6 g/L, with complete inhibition of growth above 40 g/L (Adegbola, 2008). Conversely, few studies have reported MeOH toxicity affecting the ability of *M. trichosporium* OB3b to oxidize CH_4_ under reaction conditions. Kim et al. (2010) reported a decrease in MeOH production and reduction of nearly 30% in pMMO activity with an initial 5 mM concentration (equivalent to 160 mg/L). On the contrary, another work mentioned that 400 mg/L of MeOH did not affect the enzymatic activity of MMO measured by propylene epoxidation (Hwang et al., 2015).

**FIGURE 6:**
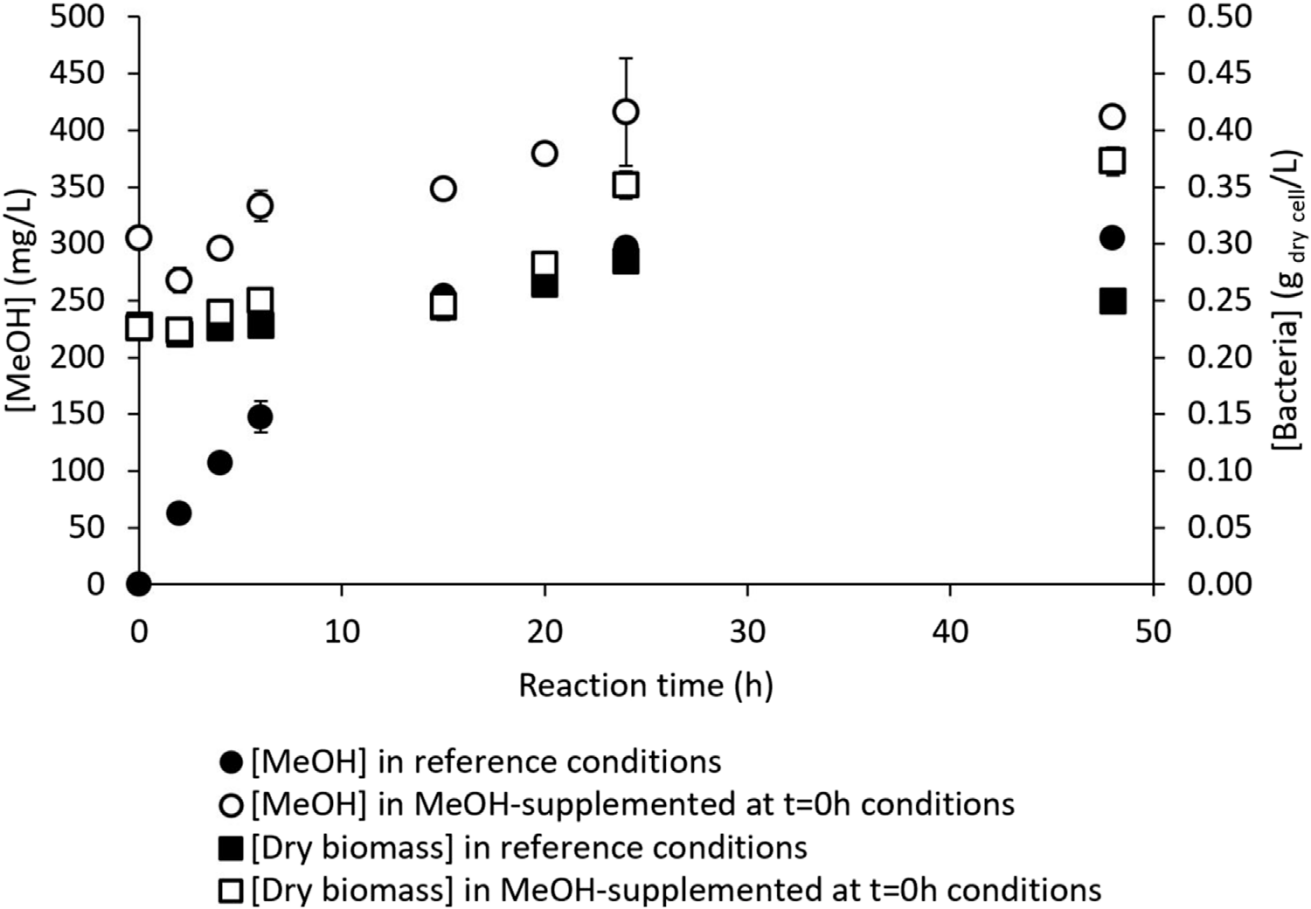
Kinetics of CH_4_ hydroxylation by *M. trichosporium* OB3b in the batch reactor in the reference reaction medium (MeOH concentration [MeOH] in filled circles; dry biomass concentration [Dry biomass] in filled squares) and in the reaction medium supplemented with 300 mg/L of MeOH at t = 0 h (MeOH concentration [MeOH] in empty circles; dry biomass concentration [Dry biomass] in empty squares).

MeOH toxicity on the ability of *M. trichosporium* OB3b to oxidize CH_4_ and produce MeOH thus needed to be clarified further. *M. trichosporium* OB3b was freshly recovered from the culture, as described in Section 2.2, and thus assumed to be in a good physiological state. The cells were pelleted and resuspended in a RM initially supplemented with the maximal concentration of MeOH reached in the previous assays (*i.e.,* 300 mg/L) to test their hydroxylating activity. A test was also conducted with the same bacterial suspension under reference conditions, *i.e.*, without any MeOH addition. Figure 6 shows the dry biomass and MeOH kinetics for both conditions. The MeOH kinetics obtained under the reference conditions were similar to those observed typically (Section 3.1); here, MeOH increased linearly over the first 6 h to 148 ± 14 mg/L, followed by slower production until reaching 297 ± 1 mg/L at 24 h, and plateauing around 305 ± 3 mg/L at t = 48 h (Figure 6). The dry biomass concentration started at 0.228 ± 0.005 g_dry cell_/L and ended at 0.250 ± 0.004 g_dry cell_/L (Figure 6), remaining generally stable along the reaction. The pH evolved from 7.00 ± 0.00 to 7.84 ± 0.04, as commonly observed.

A slight decrease in MeOH concentration from 305 ± 1 mg/L at t = 0 h to 268 ± 11 mg/L at t = 2 h was observed in the MeOH-supplemented condition (Figure 6). This indicates that the bacterial cells consumed a part of the MeOH supplied and were able to overcome MDH inhibition despite the presence of EDTA and NaCl. This finding is in accordance with a previous work carried out by our group (Pen et al., 2016), where *M. trichosporium* OB3b exhausted 120 mg/L of MeOH, with MeOH as the sole carbon source (no CH_4_ injected in the headspace), despite the addition of EDTA and NaCl. The MeOH concentration then increased to 379 ± 7 mg/L at 20 h and stabilized at around 412 ± 8 mg/L at 48 h (Figure 6). Taking into account the initially added MeOH, the amount of MeOH effectively produced and excreted by *M. trichosporium* OB3b was 0.64 ± 0.04 mg, which is far from the 1.83 ± 0.02 mg obtained under reference conditions (Figure 6).

Meanwhile, significant evolution of the dry biomass from 0.226 ± 0.003 g_dry cell/_L at t = 0 h to 0.373 ± 0.012 g_dry cell_/L at t = 48 h was observed in the MeOH-supplemented RM (Figure 6). The pH followed a trend similar to that under the reference conditions (from 7.00 ± 0.00 to 7.69 ± 0.13), probably due to both MeOH production and cell growth, suggesting that the pH was not a determining parameter in this case. MeOH addition at the beginning of the experiment did not prevent bacterial growth; *M. trichosporium* OB3b was thus able to overcome MDH inhibition to feed the carbon assimilation and biomass synthesis pathways with methanol. Metabolic regulation likely occurred to balance the MeOH accumulation induced by the MDH inhibitors and MeOH consumption supporting bacterial proliferation. This hypothesis could explain the lower MeOH production in the MeOH-supplemented condition.

In both conditions tested, the bacteria were exposed to similar MeOH concentrations of around 300 mg/L at different reaction times (t = 0 h *vs.* t = 24 h). The freshly cultivated bacteria were implemented in the MeOH-supplemented condition, whereas the bacteria endured a 24-h reaction under the reference condition. The freshly cultivated bacteria were able to consume a part of the MeOH and grow, while the bacteria which underwent the 24-h reaction did not. As a result, MeOH has no toxicity toward the cells in the conditions tested. On the contrary, the reaction conditions are likely to be deleterious as *M. trichosporium* OB3b was not able to take up the available MeOH at the end of the reaction process (from 24 h to 48 h) even after reaching a possible MeOH threshold that allowed MeOH consumption. The results of NMR analyses showed that formate was used for NADH regeneration (Section 3.2) to supply the reducing power; thus, a monitoring of the formate concentration was carried out thereafter.

### 3.6 Formate effects on methanol production

**FIGURE 7:**
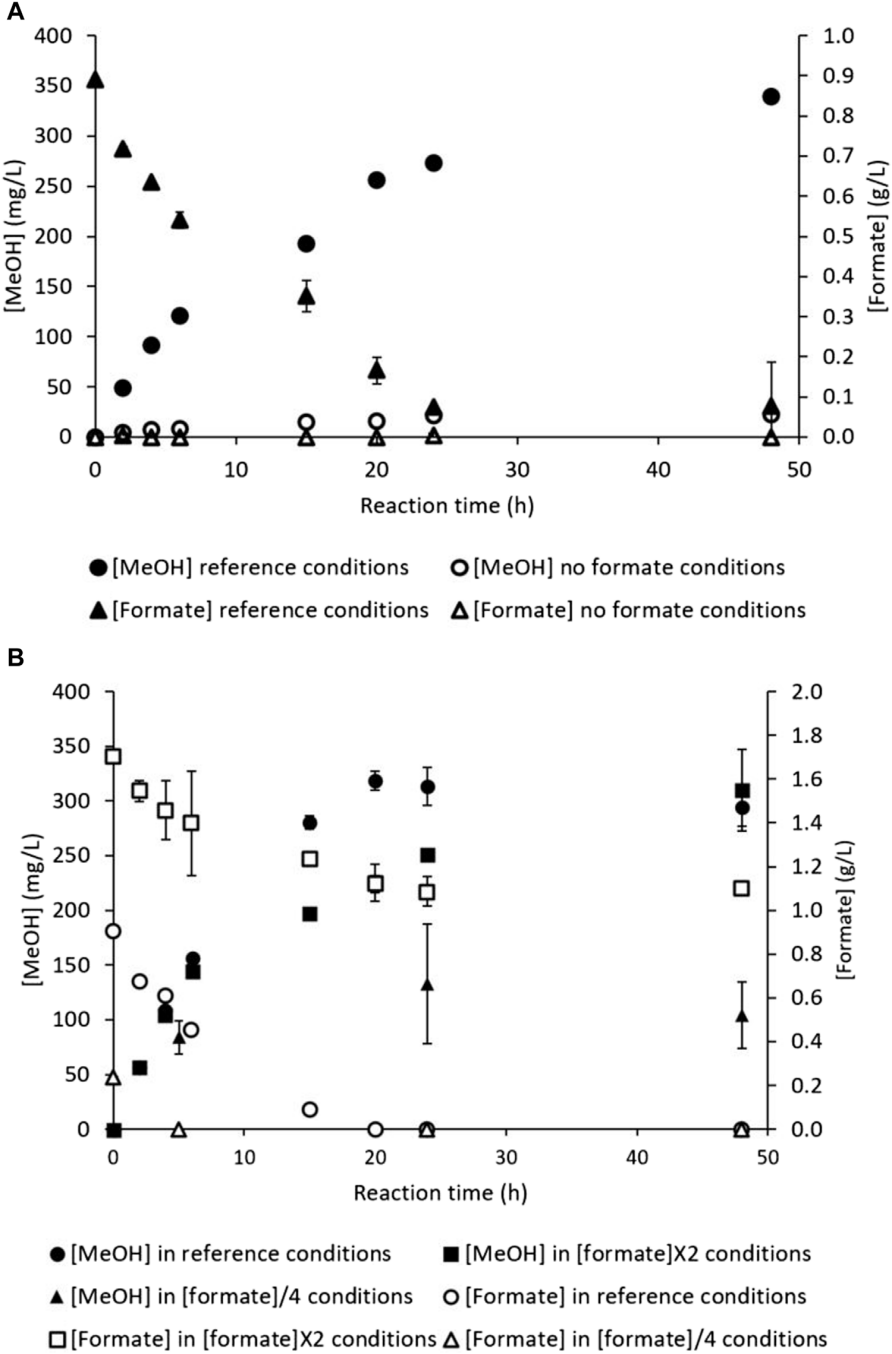
CH_4_ hydroxylation by *M. trichosporium* OB3b in the batch mode: **(A)** under the reference conditions (filled circles for MeOH concentration and filled triangles for formate concentration) and formate-free conditions (empty circles for MeOH concentration and empty triangles for formate concentration); **(B)** under the reference conditions (filled circles for MeOH concentration and empty circles for formate concentration), [formate]×2 conditions (filled squares for MeOH concentration and empty squares for formate concentration), and [formate]/4 conditions (filled triangles for MeOH concentration and empty triangles for formate concentration).

As depicted in Figure 1, formate acts as an external electron donor to counteract the stop of NADH regeneration due to MDH inhibition. The general equation for CH_4_ oxidation indicates equimolar MeOH production and formate consumption (Equation 3); these molecules should thus evolve in a 1:1 ratio. Complete formate uptake would result in a reducing power shortage (according to Section 3.2), possibly stopping MeOH production. Nevertheless, no formate monitoring has been reported in literature up to date to compare the formate consumption kinetics with MeOH production during reaction. Assays were hence carried out to measure the formate concentration during the CH_4_ hydroxylation reaction. A hydroxylation assay was first carried out under reference condition to monitor the methanol ([MeOH], mg/L) and formate ([formate], g/L) concentrations (Figure 7A). Another reaction assay implemented in parallel in the RM devoid of formate (Figure 7A) to investigate if *M. trichosporium* OB3b was able to use other electron donors, especially intracellular resources (Kalyuzhnaya, 2016).

From 0 to 24 h, the reference conditions showed the usual MeOH accumulation profile (Figure 7A); MeOH increased quasilinearly from 0 to about 275 mg/L, corresponding to 51 µmol of MeOH produced. Meanwhile, the formate concentration also decreased quasilinearly from 0.89 to about 0.07 g/L, which represents 109 µmol of formate consumed and 92% of the initial supplied quantity. Therefore, the consumed formate quantity was almost two-fold higher than the MeOH produced. According to the results described in Section 3.2, the bacterial cells mainly converted formate to CO_2_ to regenerate NADH. In the meantime (0–24 h), no MeOH production was observed in the RM devoid of formate. No formate accumulation was evidenced either, meaning bacterial cells did not produce formate in the RM (Figure 7A). Formate addition thus appears to be necessary for MeOH production. This result contradicts the findings of the study conducted by Duan et al. (2011), where MeOH concentration reached around 0.6 g/L without formate addition to the reaction medium. An external electron donor supply is thus mandatory to start CH_4_ hydroxylation.

From 24 h to 48 h, the reference conditions displayed slowed MeOH production. The MeOH concentration increased from about 275 mg/L to 340 mg/L at 48 h, which corresponds to 12 µmol of methanol produced. The formate concentration was constant and equal to 0 g/L (Figure 7A). *M. trichosporium* OB3b was hence able to accumulate a little MeOH despite formate depletion. Formate deficiency can also cause NADH regeneration stop, leading to NADH shortage. *M. trichosporium* OB3b may then switch to a previously constituted NADH stock. In the RM without formate, the MeOH and formate concentrations remained stable from 24 h to 48 h.

The dry biomass generally remained constant during reaction under both conditions, starting with an initial concentration of 0.16 ± 0.01 g_dry cell_/L at t = 0 h and ending at 0.16 ± 0.03 g_dry cell_/L at t = 48 h for the reference and formate-free RM. The absence of formate thus had no significant impact on dry biomass concentration and did not induce cell death. The pH under the reference conditions increased normally from 6.98 ± 0.00 at t = 0 h to 7.77 ± 0.05 at t = 48 h, reflecting proton consumption with concomitant MeOH production. Conversely, the pH remained quasi-constant from 6.98 ± 0.00 at t = 0 h to 6.95 ± 0.01 at t = 48 h in the formate-free condition. As *M. trichosporium* OB3b did not produce any methanol in the absence of the external electron donor, the pH of the RM did not vary. This result confirms that the increase in pH is also an indicator of CH_4_ biohydroxylation.

The intracellular resources of *M. trichosporium* OB3b did not allow commencement of MeOH production; hence, formate addition at t = 0 h was mandatory. Additional reaction experiments were implemented with a 4-fold decreased initial formate concentration ([formate]/4) or doubled initial concentration ([formate]×2) to investigate whether MeOH and formate followed a dose–effect relationship. Increasing the initial formate concentration may also be a possible method of preventing formate exhaustion. The MeOH and formate kinetics are displayed in Figure 7B. In all cases, the pH followed identical evolutions (increasing from 7.00 ± 0.00 at t = 0 h to 7.80 ± 0.03 at t = 48 h), and the dry biomass concentration remained quasi-constant (data not shown). Consequently, modifying the initial formate concentration did not affect the pH of the RM and did not promote bacterial growth during the reaction.

Under the reference conditions, quasilinear MeOH production was observed up to 318 ± 9 mg/L from 0 to 20 h, which is equivalent to 59.6 µmol of MeOH produced (Figure 7B). This was associated with an almost linear and complete consumption of the supplied formate, *i.e*., 0.9 ± 0.0 g/L, equal to 120.6 µmol of formate consumed (Figure 7B). As observed previously, a quasi-plateau of the MeOH concentration around 300 mg/L was attained at 20 h, concomitant with formate exhaustion (Figure 7B). Lowering the initial formate concentration resulted in decreased MeOH production. The MeOH production increased slowly to 84 ± 16 mg/L at t = 5 h and stabilized around 104 ± 30 mg/L at t = 48 h, far from the usual plateau at around 300 mg/L (Figure 7B). The formate concentration decreased quickly in the meantime, as formate was exhausted as early as t = 5 h (Figure 7B). The 4-fold decrease in formate concentration therefore resulted in 3-fold decrease in MeOH production. Decreasing the initial formate concentration thus affects the final MeOH concentration unfavorably, highlighting a dose–effect relationship. Therefore, formate shortage must be avoided during the reaction to maintain effective MeOH production up to 48 h. Formate exhaustion at t = 20 h under the reference condition, as seen in Figure 7A, may limit the MeOH production ability of *M. trichosporium* OB3b.

Consequently, an assay was implemented with doubled initial formate concentration ([formate]×2), which induced lower MeOH accumulation over the first 20 h than under the reference conditions (Figure 7B). The MeOH concentration reached 226 ± 17 mg/L at 20 h in the [formate]×2 condition, equal to 42.2 µmol of methanol produced (Figure 7B). Meanwhile, the formate concentration decreased from 1.7 ± 0.0 to 1.1 ± 0.0 g/L, representing a consumption of 77.4 µmol (around 34% of the initial quantity, Figure 7B). Again, the consumed formate was around twice the quantity of MeOH produced under both the reference and [formate]×2 conditions.

The MeOH production increased from 226 ± 17 at 20 h to 310 ± 38 mg/L at 48 h, equivalent to 15.8 µmol (Figure 7B), in the [formate]×2 condition. The final MeOH concentration was similar to that observed under the reference conditions, showing no significant impact of the doubled electron donor quantity on MeOH accumulation. However, the formate concentration leveled off at 1.1 ± 0.0 g/L (Figure 7B), stressing methanol production without any formate consumption. Several phenomena could explain this surprising behavior: *M. trichosporium* OB3b could have stocked sufficient quantities of regenerated NADH during the first 20 h to supply MMO until the end of reaction, or the strain may have switched to an intracellular electron donor to pursue MeOH accumulation.

To summarize, doubling the initial formate concentration had no significant impact on the final MeOH concentration but slightly influenced the production kinetics (MeOH accumulation kinetics were slightly slowed in the [formate]×2 condition). On the contrary, formate uptake appears to be regulated by the initial formate concentration in the RM. These results are in agreement with a previous assay carried out at our laboratory (Pen et al., 2016), where a formate pulse containing the same amount of formate as that added to the RM initially was applied during reaction at 4.5 h (*i.e.*, during the linear MeOH production phase). This formate pulse reduced MeOH production compared to the reference condition, suggesting a regulation process (Pen et al., 2016). An additional assay was implemented here: a formate pulse was supplied at t = 5 h. However, this addition was meant to replenish the formate quantity to its initial value. A 50× sodium formate solution was used to ensure negligible dilution of the reaction mixture. Table 1 displays the MeOH and formate quantifications at 24 h.

**TABLE 1:** CH_4_ hydroxylation by *M. trichosporium* OB3b in the batch mode under the reference conditions or with a formate pulse during the reaction to replenish the formate content to its initial value (at t = 5 h). The methanol concentrations were quantified at t = 24 h.

	Reference conditions	Formate pulse conditions
Final [MeOH] (mg/L)	261.2 ± 12.3	274.0 ± 53.4
Final [Formate] (g/L)	0.00 ± 0.00	0.15 ± 0.00
Final [Biomass] (g_dry cell_/L)	0.352 ± 0.007	0.355 ± 0.002

At t = 24 h, the final MeOH concentration reached 261 ± 12 mg/L under the reference conditions and 274 ± 53 mg/L with the formate pulse (Table 1). Thus, the MeOH production did not differ significantly after formate addition. The dry biomass also showed a similar trend, with 0.352 ± 0.007 g_dry cell_/L under the reference conditions against 0.355 ± 0.002 g_dry cell_/L with the formate pulse (Table 1). Therefore, formate supplementation during the reaction neither improved MeOH production nor influenced bacterial proliferation. Moreover, the bacterial cells did not consume all of the supplied formate (Table 1). Hence, the MeOH production stop was not caused by formate shortage during the reaction but rather by the limited bacterial hydroxylating capacity because of physiological stress affecting the bacteria.

## 4 Conclusion

MeOH production stop by *M. trichosporium* OB3b in the batch mode was investigated in this work. There were no observed limitations from gaseous substrates or nutrients during the reactions. The results of ^13^C-NMR analyses showed that MeOH was produced only by direct CH_4_ oxidation without formate reduction. In the conditions tested in this work, MeOH was not cytotoxic to the bacteria, whereas the reaction conditions generally appeared to be stressful. Formate was mainly used as an electron donor for NADH regeneration. However, the mass balance calculations highlighted that only half of the NADH produced was devoted to MeOH production. Increasing the formate concentration did not improve MeOH production, hinting that there was metabolic regulation. Such biological phenomena need to be investigated further to possibly extend the methanol production time of *M. trichosporium* OB3b.

## Data Availability

The raw data supporting the conclusions of this article will be made available by the authors without undue reservation.
