# Supplementary material for: Methane biohydroxylation into methanol by Methylosinus trichosporium OB3b: possible limitations and formate use during reaction
Source: Front Bioeng Biotechnol. 2024 Aug 26;12:1422580. doi: 10.3389/fbioe.2024.1422580 (PMC11381948; doi:10.3389/fbioe.2024.1422580)
Supplement: Supplementary file 1 [file Image1.pdf]

Supplementary Material

1 Supplementary Data

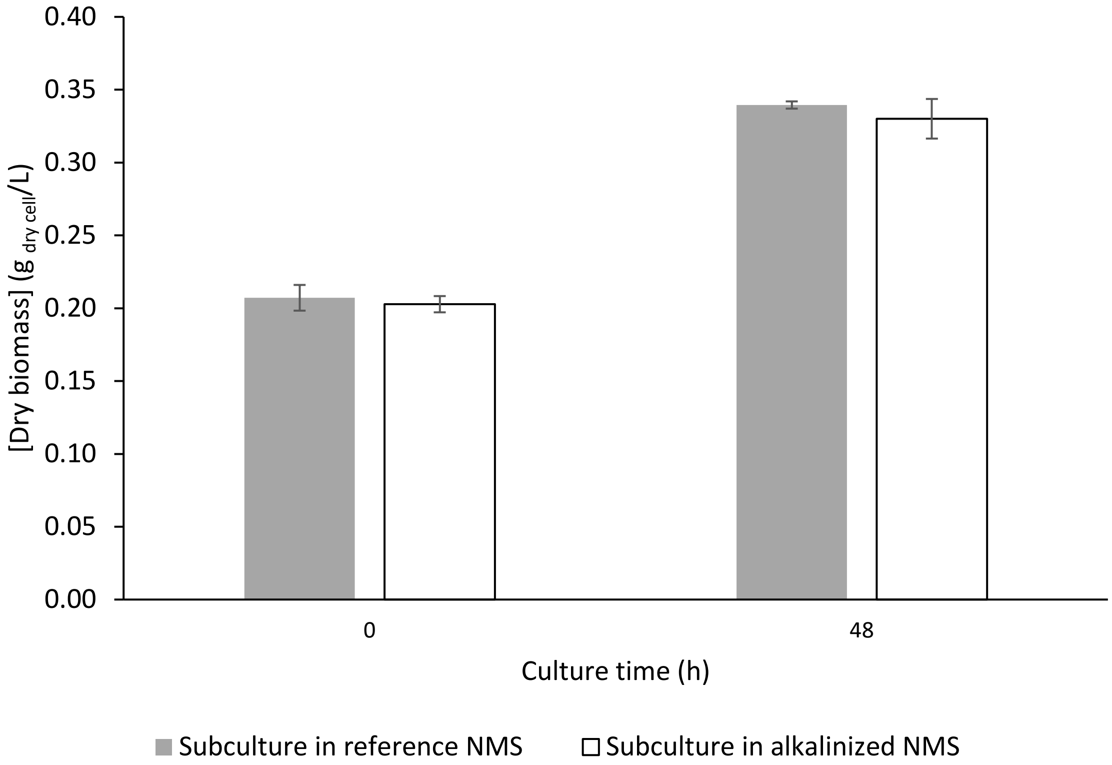

SM1: *Methylosinus trichosporium* OB3b growth tests: subcultures in reference NMS (pH adjusted to 7.0, filled bars) or alkalinized NMS (pH adjusted to 7.85, empty bars)
